# Ferulic Acid Attenuates the Injury-Induced Decrease of Protein Phosphatase 2A Subunit B in Ischemic Brain Injury

**DOI:** 10.1371/journal.pone.0054217

**Published:** 2013-01-17

**Authors:** Phil-Ok Koh

**Affiliations:** Department of Anatomy, College of Veterinary Medicine, Research Institute of Life Science, Gyeongsang National University, Jinju, South Korea; Institut National de la Santé et de la Recherche Médicale (INSERM U901), France

## Abstract

**Background:**

Ferulic acid provides a neuroprotective effect during cerebral ischemia through its anti-oxidant function. Protein phosphatase 2A (PP2A) is a serine and threonine phosphatase that contributes broadly to normal brain function. This study investigated whether ferulic acid regulates PP2A subunit B in a middle cerebral artery occlusion (MCAO) animal model and glutamate toxicity-induced neuronal cell death.

**Methodology/Principal Findings:**

MCAO was surgically induced to yield permanent cerebral ischemic injury in rats. The rats were treated with either vehicle or ferulic acid (100 mg/kg, i.v.) immediately after MCAO, and cerebral cortex tissues were collected 24 h after MCAO. A proteomics approach, RT-PCR, and Western blot analyses performed to identification of PP2A subunit B expression levels. Ferulic acid significantly reduced the MCAO-induced infarct volume of the cerebral cortex. A proteomics approach elucidated the reduction of PP2A subunit B in MCAO-induced animals, and ferulic acid treatment prevented the injury-induced reduction in PP2A subunit B levels. RT-PCR and Western blot analyses also showed that ferulic acid treatment attenuates the injury-induced decrease in PP2A subunit B levels. Moreover, the number of PP2A subunit B-positive cells was reduced in MCAO-induced animals, and ferulic acid prevented these decreases. In cultured neuronal cells, ferulic acid treatment protected cells against glutamate toxicity and prevented the glutamate-induced decrease in PP2A subunit B.

**Conclusions/Significance:**

These results suggest that the maintenance of PP2A subunit B by ferulic acid in ischemic brain injury plays an important role for the neuroprotective function of ferulic acid.

## Introduction

Cerebral ischemia is the most common cause of stroke. Cerebral ischemia leads to production of free radicals such as reactive oxygen species (ROS) and activates inflammatory response. Ferulic acid (4-hydroxy-3-methoxycinnamic acid), a constituent of *Anglica sinensis (olivi) Didl.* and *Ligusticum chuoanxiong Hort.,* eliminates free radicals and exerts anti-inflammatory activity animals [1], [2], [3], [4]. Moreover, ferulic acid provides neuroprotective effects against transient focal cerebral ischemia in experimental animals [1], [2].

Protein phosphatase 2A (PP2A) is an essential serine and threonine phosphatase protein which is involved in the regulation of several cellular functions including cell differentiation, apoptosis, and signal transduction [5], [6], [7], [8]. PP2A is a trimeric protein complex that is composed of a scaffolding subunit A, a variable regulatory subunit B, and a catalytic subunit C [8], [9]. The structural A subunit dimerizes with the catalytic C subunit and binds to regulatory B subunits [7]. Moreover, the substrate specificity of PP2A is conferred by the regulatory B subunit [10]. The A and C subunits of PP2A are ubiquitously expressed in most tissues, while subunit B is highly enriched in the brain [7], [11]. Thus, it is accepted that the regulatory subunit B modulates various functions of PP2A and performs an essential role in the nervous system [11], [12]. We previously reported on both the decrease of PP2A subunit B after cerebral ischemic injury and the neuroprotective effect of ferulic acid against ischemic brain injury [13], [14]. We hypothesize that ferulic acid may contribute to the regulation of PP2A subunit B expression in ischemic brain injury. Thus, this study investigates whether ferulic acid can modulate the expression level of PP2A subunit B during cerebral ischemic injury and glutamate-induced neuronal cells damage.

## Results

**Figure 1:**
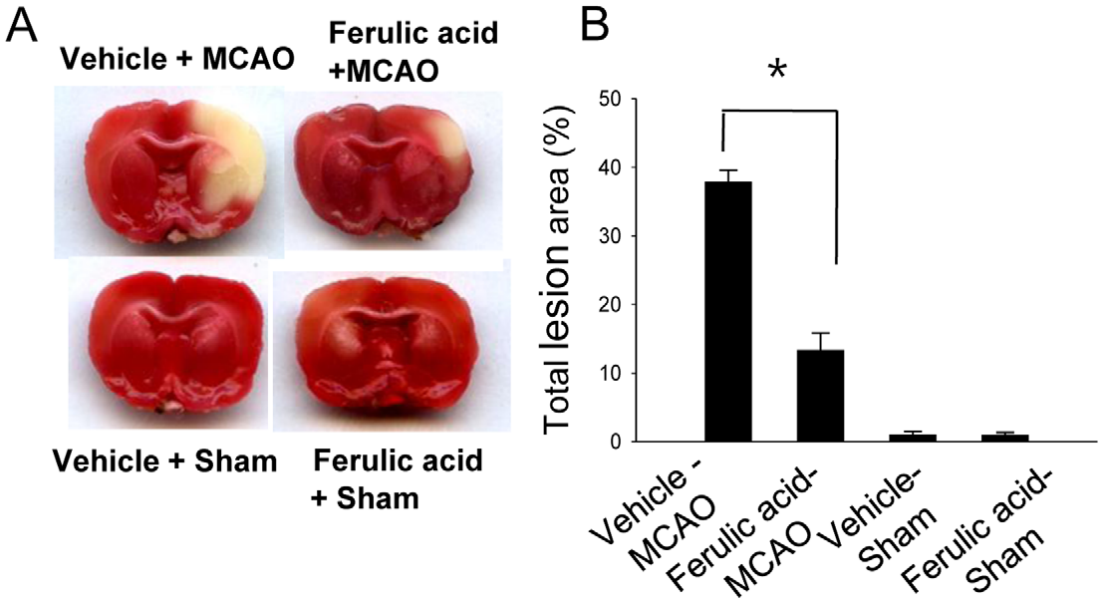
The neuroprotective effect of ferulic acid in focal cerebral ischemia. Representative photos of TTC stain in the cerebral cortices of vehicle+sham, ferulic acid+sham, vehicle+middle cerebral artery occlusion (MCAO), ferulic acid+MCAO animals. Animals were treated with vehicle or ferulic acid prior to MCAO. Brain sections were stained by TTC (A). The ischemic area remained white, while the intact area was stained red. The percentage of ischemic lesion area was calculated by the ratio of the infarction area to the whole slice area (B). Data are means ± S.E.M. * *P*<0.05.

**Figure 2:**
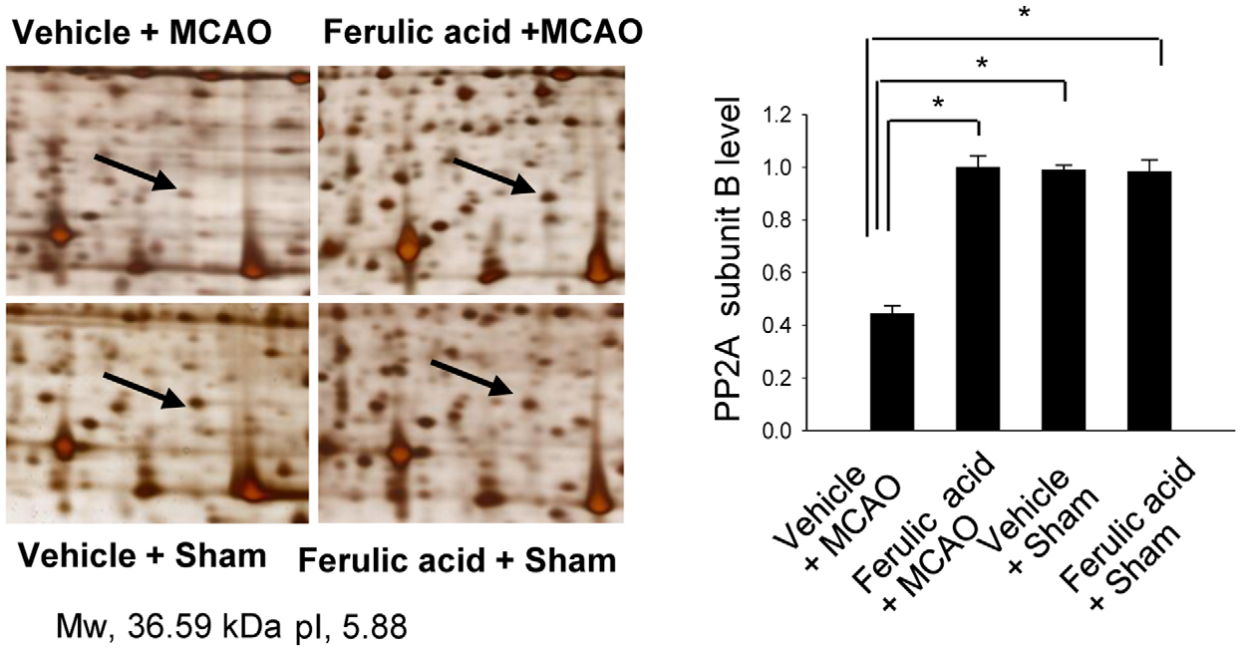
The change of protein phosphatase 2A (PP2A) subunit B protein spots by ferulic acid in focal cerebral ischemia. Protein phosphatase 2A (PP2A) subunit B protein spots identified by MALDI-TOF in the cerebral cortices from vehicle+MCAO, ferulic acid+MCAO, vehicle+sham, ferulic acid+sham animals. Animals were treated with vehicle or ferulic acid prior to MCAO. Arrows indicate the protein spots. Mw and pI indicate molecular weight and isoelectrical point, respectively. The intensity of spots was measured using PDQuest software. The ratio of intensity is described as spots intensity of these animals to spots intensity of sham+vehicle animals. Data are shown as mean ± S.E.M. * *P*<0.05.

We confirmed the neuroprotective effect of ferulic acid against cerebral ischemic injury using TTC staining (Fig. 1). Ferulic acid treatment significantly decreased the volume of infarct regions during MCAO compared with those of the vehicle-treated animals. Proteomics identified the PP2A subunit B protein spots as differentially expressed proteins in the cerebral cortices of vehicle+MCAO and ferulic acid+MCAO animals (Fig. 2). The peptide mass of the PP2A subunit B is 9/56, and the sequence of this protein is 29%. PP2A subunit B levels were significantly decreased in vehicle+MCAO animals relative to sham-operated animals, whereas expression of PP2A subunit B was attenuated in ferulic acid+MCAO animals. PP2A subunit B levels were 0.45±0.02 and 0.96±0.03 in vehicle- and ferulic acid-treated animals during MCAO, respectively.

**Figure 3:**
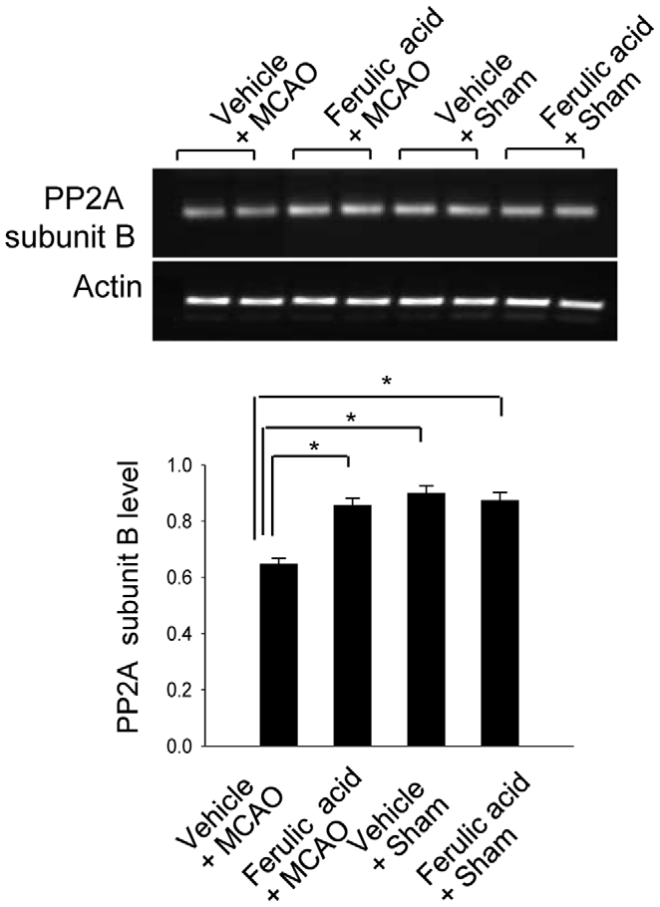
The change of protein phosphatase 2A (PP2A) subunit B protein mRNA by ferulic acid in focal cerebral ischemia. RT-PCR analysis of protein phosphatase 2A (PP2A) subunit B in the cerebral cortices from vehicle+MCAO, ferulic acid+MCAO, vehicle+sham, ferulic acid+sham animals. Animals were treated with vehicle or ferulic acid prior to MCAO. Each lane represents an individual experimental animal. Densitometric analysis is represented as intensity of PP2A subunit B to intensity of actin. Data (*n* = 5) are represented as mean ± S.E.M. * *P*<0.05.

**Figure 4:**
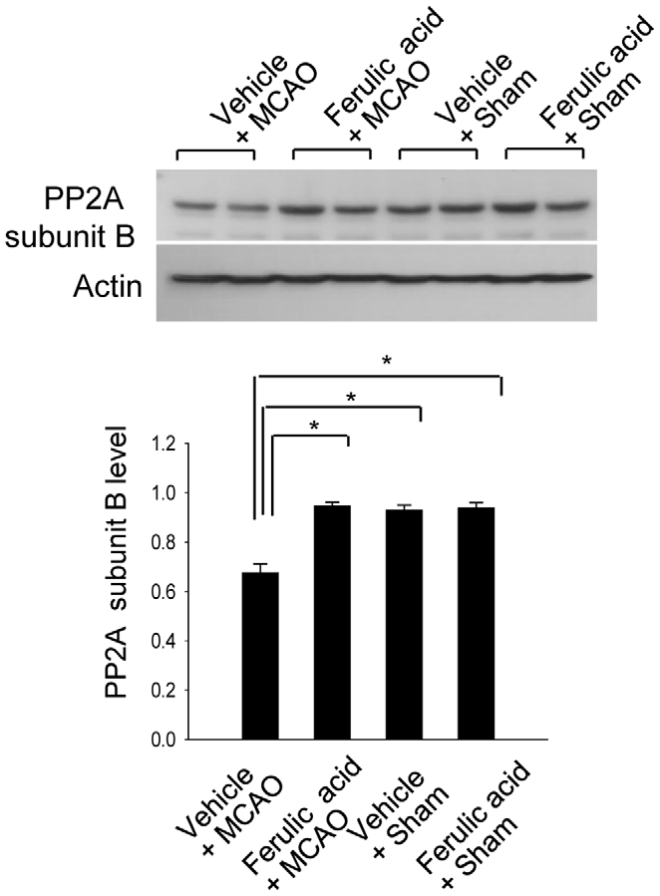
The change of protein phosphatase 2A (PP2A) subunit B protein by ferulic acid in focal cerebral ischemia. Western blot analysis of protein phosphatase 2A (PP2A) subunit B in the cerebral cortices from vehicle+MCAO, ferulic acid+MCAO, vehicle+sham, ferulic acid+sham animals. Animals were treated with vehicle or ferulic acid prior to MCAO. Each lane represents an individual experimental animal. Densitometric analysis is represented as intensity of PP2A subunit B to intensity of actin. Data (*n* = 5) are represented as mean ± S.E.M. * *P*<0.05.

**Figure 5:**
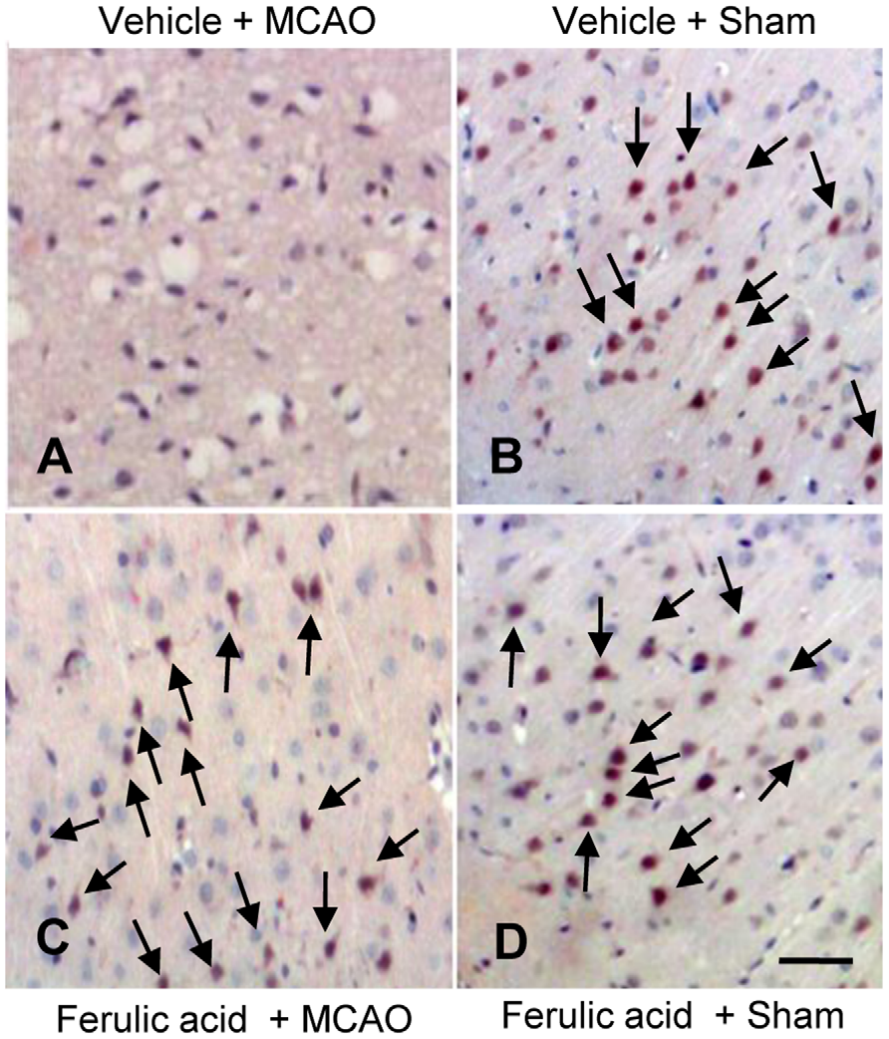
The expression of protein phosphatase 2A (PP2A) subunit B protein by ferulic acid in focal cerebral ischemia. Immuno-staining of protein phosphatase 2A (PP2A) subunit B in the cerebral cortices from vehicle+MCAO, ferulic acid+MCAO, vehicle+sham, ferulic acid+sham animals. Animals were treated with vehicle or ferulic acid prior to MCAO. Arrows indicate positive cells of PP2A subunit B. Scale bar = 100 μm.

RT-PCR and Western blot analyses clearly showed the changes in PP2A subunit B levels induced by ferulic acid treatment. In result of RT-PCR, levels of PP2A subunit B were 0.63±0.02 and 0.82±0.02 in the cerebral cortices of vehicle- and ferulic acid-treated, MCAO-operated animals, respectively (Fig. 3). Moreover, PP2A subunit B protein levels were decreased in vehicle+MCAO animals compared to sham-operated controls, whereas ferulic acid treatment prevented the injury-induced reduction in PP2A subunit B protein levels. PP2A subunit B protein levels were 0.71±0.02 and 0.89±0.02 in the cerebral cortices of vehicle- and ferulic acid-treated animals during MCAO, respectively (Fig. 4). The numbers of cells positive for PP2A subunit B were decreased in the cerebral cortices of vehicle+MCAO animals relative to sham-operated animals. Ferulic acid prevented the injury-induced decreases in the numbers of positive cells for PP2A subunit B (Fig. 5).

**Figure 6:**
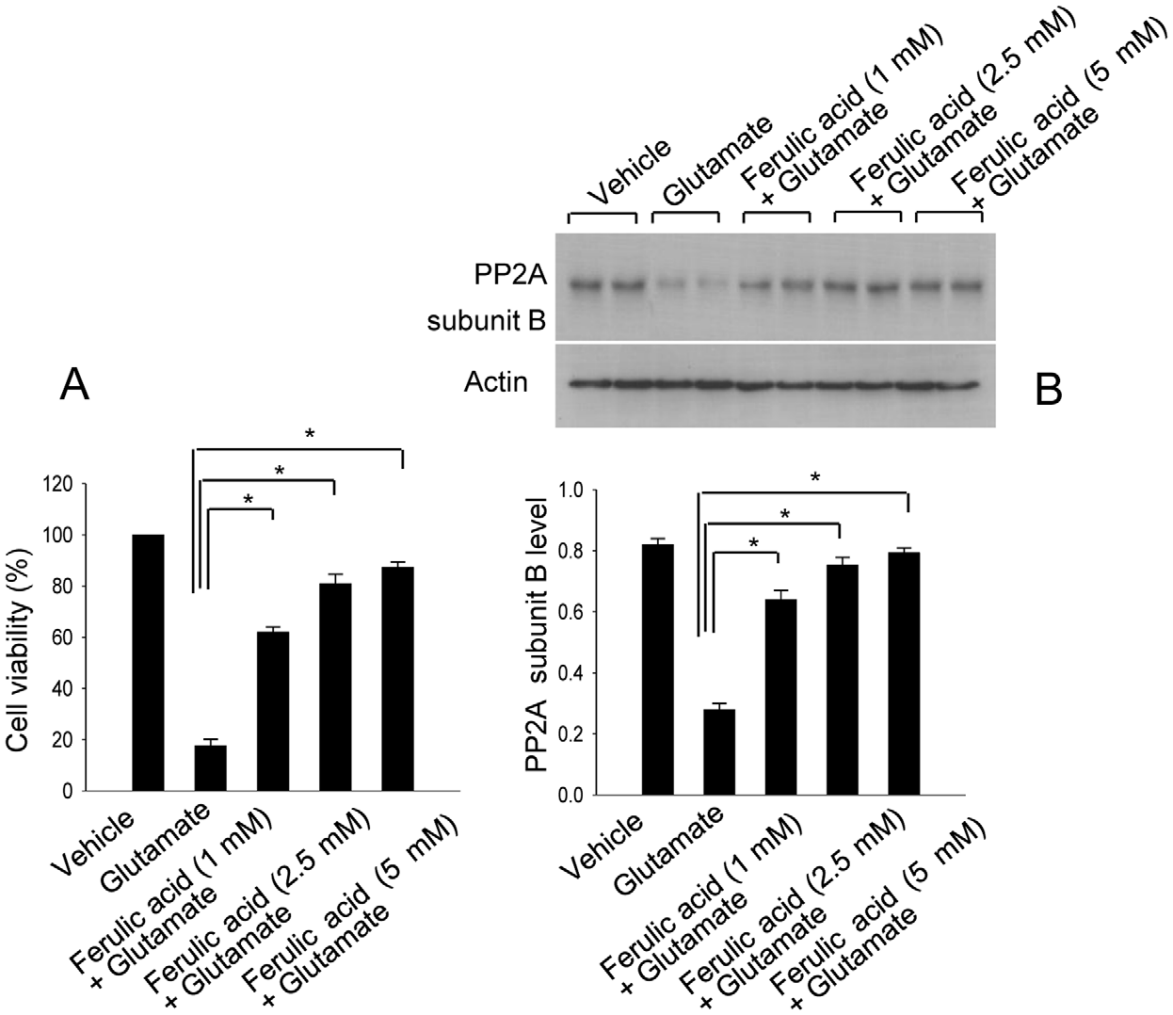
The neuroprotective effect of ferulic acid and protein phosphatase 2A (PP2A) subunit B protein expression in HT22 cells. Cell viability (A) and Western blot analysis of protein phosphatase 2A (PP2A) subunit B (B) in HT22 cells. Glutamate (5 mM) was exposed to HT22 cells for 24 h and ferulic acid (1, 2.5, 5 mM) was treated at 30 min before glutamate exposure. Cellular viability was assessed using the MTT assay (A). Cell survival was expressed as percentage of neuroprotection vs. vehicle set at 100%. Densitometric analysis is represented as intensity of PP2A subunit B to intensity of actin (B). Data (*n* = 5) are represented as mean ± S.E.M. * *P*<0.05.

The neuroproective effect of ferulic acid following glutamate exposure was confirmed using MTT assay (Fig. 6A). PP2A subunit B levels were significantly decreased in glutamate-exposed cells, whereas ferulic acid treatment attenuated this glutamate-induced decrease in a dose-dependent manner (Fig. 6B). PP2A subunit B levels were 0.27±0.02 in the glutamate-treated group, and 0.64±0.03, 0.75±0.02, and 0.79±0.01 in the ferulic acid-treated groups (1, 2.5, 5 mM of ferulic acid, respectively).

## Discussion

Ferulic acid exerts a neuroprotective function following transient focal cerebral ischemia through its anti-oxidant and anti-inflammatory effects [1], [2]. We previously demonstrated that ferulic acid decreases infarct regions and attenuates neuronal cell death by modulating cell survival and apoptosis signaling pathways in cerebral ischemia [14.15]. In the present study, the results of TTC staining also demonstrate the neuroprotective effect of ferulic acid in an MCAO-induced animal model. We previously demonstrated that ferulic acid contributes to the up- and down-regulation of various specific proteins during MCAO injury. Among these proteins, this study was focused on PP2A subunit B expression [16].

PP2A can regulate cellular activity including cell development and apoptosis [5], [6], [7], [17]. Moreover, it is accepted that PP2A can regulate normal brain function [12]. Thus, the reduction of PP2A causes central nervous system disorders such as Alzheimer’s disease [18]. PP2A dephosphorylates the microtubule-associated protein tau that stabilizes microtubule assembly, especially in the axon. However, a decrease of PP2A activity induces the hyperphosphorylation of tau protein [18], [19], and hyperphosphorylated tau leads to axonal degeneration and results in neuronal disorder [20]. Previous studies demonstrated that hyperphosphorylated tau is associated with decreases in PP2A level [18], [19]. Thus, the maintenance of PP2A level is a critical event in the regulation of some physiological neuronal cell functions. Among PP2A subunits, the regulatory B subunit exists abundantly in brain tissue and modulates various functions of PP2A in the brain. It is accepted that the substrate specificity of PP2A is regulated by the regulatory B subunit [10]. Previous studies demonstrated that overexpression of PP2A prevents hydrogen peroxide -activation of Erk/12, JNK and p38, and inhibits apoptosis [21]. Moreover, the inhibition of protein phosphatase 2A B subunit by E4orf4 protein leads to G2/M cell cycle arrest and enhances cell death [22]. This study clearly showed the decreases of PP2A subunit B in MCAO- and glutamate-induced damages. These data can demonstrate that maintenance of PP2A is a critical factor for the cell survival. Our proteomics approach clearly demonstrated that ferulic acid treatment prevents the MCAO-induced decrease of PP2A subunit B. Moreover, this study clearly assessed the regulation of PP2A subunit B expression by ferulic acid during focal cerebral ischemia using RT-PCR and Western blot analyses. MCAO induces the reduction of PP2A subunit B expression in the cerebral cortex, and ferulic acid treatment prevents this injury-induced decrease in PP2A subunit B expression. In addition, immunohistochemical staining confirmed that ischemic brain injury decreases the numbers of PP2A subunit B positive cells, whereas ferulic acid attenuates the injury-induced decrease. Decreased PP2A subunit B expression can permit the phosphorylation of tau and result in neuronal cell damage. Ferulic acid prevents a down-regulation of PP2A subunit B in cerebral ischemic injury and attenuates neuronal cell death. Thus, these results demonstrate that ferulic acid regulates PP2A subunit B expression in focal cerebral ischemic injury.

In cultured hippocampal cells, glutamate treatment induces oxidative stress and leads to neuronal cell death [23]. We previously reported the neuroprotective effect of ferulic acid against glutamate exposure-induced cell death [14], [15]. Moreover, the present study showed that glutamate exposure induces a reduction of PP2A subunit B expression;,whereas ferulic acid prevents the glutamate-induced decrease in PP2A subunit B expression. The effect of ferulic acid appears to be dose-dependent. This study clearly demonstrates that ferulic acid treatment modulates PP2A subunit B expression in neuronal cells injury using both *in vivo* and *in vitro* models.

### Conclusions

Our results support the hypothesis that the maintenance of PP2A subunit B by ferulic acid in ischemic brain injury ultimately leads to reduction of neuronal cell death. Thus, this study suggests that ferulic acid treatment reduces neuronal cell damage from ischemic brain injury by modulating PP2A subunit B expression.

## Materials and Methods

### Animals and Melatonin Treatment

Male Sprague-Dawley rats weighing 210∼230 g (*n* = 40) were kept under temperature (25°C) and lighting (12/12 light/dark cycle). All experimental protocols for animal use were approved by Institutional Animal Care and Use Committee at Gyeongsang National University (Approval Number: GNU-LA-13). Animals were randomly divided four groups as follows: vehicle+sham group, ferulic acid+sham group, vehicle+middle cerebral artery occlusion (MCAO) group, and ferulic acid+MCAO group (*n* = 10 per group). Ferulic acid (Sigma, St. Louis, MO, USA) was dissolved in normal saline containing as the vehicle and 100 mg/kg of ferulic acid was injected intravenously immediately when MCAO began [1].

### Middle Cerebral Artery Occlusion

The middle cerebral artery occlusion was performed as previously described with some modification [24]. Animals were anesthetized with sodium pentobarbital (30 mg/kg, intraperitoneal injection). The right common carotid artery was exposed and the external carotid artery was cut. A 4/0 nylon filament with rounded tip by heating was inserted from the external carotid artery to internal carotid artery, advanced until the origin of the right MCA was occluded. After 24 h after the onset of occlusion, brain tissues were collected. For triphenyltetrazolium chloride (TTC) staining, brain tissues were cut into coronal slices of 2 mm in thickness. These tissues were stained for 20 min in a 2% TTC (Sigma, St. Louis, MO, USA) solution and fixed in 10% formalin. The stained tissues were photographed by a Nikon CoolPIX990 digital camera (Nikon, Tokyo, Japan) and measured for the ischemic lesion by Image-ProPlus 4.0 software (Media Cybernetics, Silver Spring, MD, USA). The ischemic lesion percentage of each slice was calculated by the ratio of the infarction area to the whole slice area.

### Two-dimensional Gel Electrophoresis, Image Analysis, and Protein Identification

Protein sample for two-dimensional gel electrophoresis was carried out using right cerebral cortices. Tissues were dissolved in lysis buffer (8 M urea, 4% CHAPS, ampholytes and 40 mM Tris-HCl) and centrigued at 16,000 g for 20 min at 4°C. The supernatant was isolated and Bradford assay (Bio-Rad, Hercules, CA, USA) was used to determine protein concentration included in supernatant. Rehydrated IPG strips (range pH 4–7 and pH 6–9, 17 cm, Bio-Rad) in sample buffer (8 M urea, 2% CHAPS, 20 mM DTT, 0.5% IPG buffer, bromophenol blue) was prepared to perform the isoelectric focusing (IEF) for 13 h at 20°C. Each protein samples was loaded in sample cup for IEF. IEF steps are described as follows: 250 V (15 min), 10000 V (3 h) and then 10000 V to 50000 V at 20°C using Protean IEF Cell (Bio-Rad). After IEF, electrophoresis was performed with gradient gel (7.5–17.5%) at 10 mA/gel for 10 h at 10°C (protein-II XI, Bio-Rad). The gels were stained with silver stain solution (0.2% silver nitrate, 0.75 mL/L formaldehyde) and were scanned using Agfar ARCUS 1200™ (Agfar-Gevaert, Mortsel, BEL). The PDQuest 2-D analysis software (Bio-Rad) was matched, analyzed, and visualized protein spots. Protein spots were subjected to trypsin digestion and peptides were extracted using extraction buffer (5% trifluoroacetic acid in 50% acetonitrile) and the extracted peptides were dried using a vacuum centrifuge for 20 min. The protein samples were analyzed by a Voyager-DE™ STR biospectrometry workstation (Applied Biosystem, Forster City, CA, USA). The database searches were carried out using MS-Fit and ProFound program. SWISS-PROT and NCBI were used as the protein sequence databases.

### RNA Isolation and Reverse Transcription-PCR Amplification

Total RNA was extracted using Trizol reagent according to the manufacturer’s instruction (Invitrogen, Carlsbad, CA, USA) and RNA (1 µg) was reverse transcribed using superscript III first-strand system for RT-PCR (Invitrogen) based on the manufacturer’s protocol. The primers sequences of PP2A subunit B and actin are 5′-CCTGGTATGCCAAACTCGAT-3′ (forward primer, PP2A subunit B), 5′- ACAATAGCCACCTGGTCGTC-3′ (reverse primer, PP2A subunit B), 5′-GGGTCAGAAGGACTCCTACG-3′ (forward primer, actin), and 5′-GGTCTCAAACATGATCTGGG-3′ (reverse primer, actin). The PCR reaction was carried out as followed: 5 min at 94°C, 30 sec at 94°C, 30 sec at 54°C, 1 min at 72°C and 10 min at 72°C. The samples were amplified 30 cycles. PCR product was run in a 1% agarose gel and visualized under UV light.

### Western Blot Analysis

The right cerebral cortices were homogenized in lysis buffer [1% Triton X-100, 1 mM EDTA in PBS (pH 7.4)] containing 10 mΜ leupeptin and 200 µM phenylmethylsulfonyl fluoride. Homogenates were centrifuged at 15,000 g for 20 min at 4°C and the supernatants were collected. Protein (30 µg) from each sample was loaded on 10% SDS–polyacrylamide gel electrophoresis. The gels were electrophoresed and transferred on the poly-vinylidene fluoride membranes (Millipore, Billerica, MA, USA). The membranes were washed in Tris-buffered saline containing 0.1% Tween-20 (TBST) and then incubated with the following antibodies: anti-PP2A subunit B antibody (diluted 1∶1,000, Cell Signaling Technology, Beverly, MA, USA) and anti-actin (diluted 1∶1,000, Santa Cruz Biotechnology, Santa Cruz, CA, USA) as primary antibody. After washing with TBST, the membrane was incubated with horseradish perxoxidase-conjugated goat anti-rabbit IgG (1∶5,000; Pierce) and the ECL Western blot analysis system (Amersham Pharmacia Biotech, Piscataway, NJ, USA) was employed to detect the signals.

### Immunohistochemical Staining

Brain tissues were fixed in 4% paraformaldehyde in 0.1 M phosphate-buffered saline (PBS, pH 7.4) and embedded in paraffin. The paraffin sections were washed with PBS, blocked with 1% normal goat serum in PBS for 1 h, and then reacted with anti-PP2A subunit B antibody (diluted 1∶50, Cell Signaling Technology) at 4°C for 15 h. After rinse with PBS, sections were reacted with biotin-conjugated goat anti-rabbit IgG (1∶200 in PBS) for 1 h, followed by incubation with an avidin-biotin-peroxidase complex for 1 h from a Vector ABC *Elite* kit (Vector Laboratories Inc., Burlingame, CA, USA). Sections were rinsed with PBS and stained with diaminobenzidine tetrahydrochloride (Sigma) solution with 0.03% hydrogen peroxidase for 3 min. Sections were counterstained with hematoxylin and dehydrated in graded alcohol. Slides were observed under a microscope and then photographed.

### Cell Culture and Treatment

Mouse hippocampal cell lines (HT22) were obtained from Dr. Noh as a gift [25] and routinely cultured in Dulbecco’s modified Eagle’s medium (DMEM, without L-glutamine) with 10% fetal bovine serum, streptomycin (100 μg/ml), and penicillin (100 unit/ml) (Gibco BRL, Gaithersburg, MD, USA). The cells were maintained in a humidified incubator with 5% CO_2_ at 37°C. HT22 cells were seeded on 60-mm culture dishes at 100,000 cells per dish. Cell density was monitored to attenuate excessive growth and was maintained 70% or less confluency [23]. Glutamate (Sigma) was treated with a final concentration of 5 mM in culture medium and cells were incubated for 24 h. Ferulic acid (1, 2.5, 5 mM) was treated at 1 h before glutamate addition. The cell viability was evaluated via an 3-(4,5-dimethylthiazol-2-yl)-2,5-diphenyltetrazoliumbromide (MTT) reduction assay. MTT solution (5 mg/ml) was added to the culture medium and cells were allowed to be maintained for 4 h at 37°C. After the remove of MTT solution, solubilization solution containing 20% sodium dodecyl sulfate (pH 4.8) and 50% dimethylformamide was added. Absorption valves at 570 nm were measured and cell viability was determined as percentage of neuroprotection vs. vehicle set at 100%.

### Statistical Analysis

All data are expressed as mean ± S.E.M. The results in each group were compared by One-way analysis of variance (ANOVA) followed by to *post-hoc* Scheffe’s test. A *P*<0.05 was considered to represent statistical significance.
